# Glucose activates the primordial follicle through the AMPK/mTOR signaling pathway

**DOI:** 10.1002/ctm2.122

**Published:** 2020-07-13

**Authors:** Shengyu Xu, Xiaoling Wu, Yanpeng Dong, Mengmeng Xu, Zimei Li, Sirun Chen, Yong Zhuo, Yan Lin, Lianqiang Che, Zhengfeng Fang, Bin Feng, Jian Li, Jianping Wang, De Wu, Zhihua Ren

**Affiliations:** ^1^ Animal Nutrition Institute, Sichuan Agricultural University; Key Laboratory of Animal Disease‐resistant Nutrition, Ministry of Education Ministry of Agriculture and Rural Affairs, Sichuan Province Chengdu Sichuan P. R. China; ^2^ College of Veterinary Medicine, Sichuan Province Key Laboratory of Animal Disease and Human Health, Key Laboratory of Environmental Hazard and Human Health of Sichuan Province Sichuan Agricultural University Chengdu Sichuan P. R. China

**Keywords:** AMPK, glucose, Hippo signaling pathway, mTOR signaling pathway, ovary culture in vitro, primordial follicle activation

## Abstract

**Background:**

We have previously found that the energy level in sows affects the activation of primordial follicles. Glucose is the primary metabolic substrate of dietary energy and its effect and mechanism of action with regards to the activation and development of primordial follicle remain unclear. Studies utilizing several different animal cells have shown that energy stress, induced by glucose starvation, activates AMPK and participates in a variety of cellular processes by regulating the Hippo and mTOR signaling pathways. However, whether glucose can affect primordial follicle activation through the above pathways has not been reported.

**Methods:**

We developed an in vitro culture system for mouse ovaries to investigate the effects of glucose on the primordial follicle activation. Protein expression of AMPK‐Hippo‐YAP and AMPK‐mTOR pathway was investigated under glucose starvation and optimal glucose level treatment. Then, ovaries were treated with AICAR or Compound C in vitro to explore the effect of AMPK activation or inhibition on primordial follicle activation, and the changes of AMPK‐Hippo‐YAP and AMPK‐mTOR signaling pathways. Finally, investigated the signaling pathways affected by glucose potentially affecting the primordial follicle activation in vivo.

**Results:**

The glucose was an essential nutrient for primordial follicle activation and we identified 25 mM glucose as the optimal level (*P* < .05) for the primordial follicle activation in vitro. The glycolysis pathway was involved in primordial follicle activation (*P* < .05) of ovaries cultured in vitro. The glucose affected the activation of primordial follicles in vitro through AMPK/mTOR signaling pathway by AMPK activation or inhibition treatment and follicle ratio count (*P* < .05). Moreover, glucose affected the primordial follicle activation of ovary in vivo via mTOR signaling pathway.

**Conclusions:**

This study demonstrates that glucose affects the primordial follicle activation through the AMPK/mTOR rather than the AMPK/Hippo signaling pathway.

**Abbreviations**
AICAR: 5‐aminoimidazole‐4‐carboxamide1‐β‐D‐ribofuranoside
AMPK: AMP‐activated protein kinase
ATP: adenosine triphosphate
COCs: cumulus‐oocyte complexes
DMSO: dimethyl sulfoxide
Lats1/2: large tumor suppressor1/2
Mst1/2: mammalian Sterile20‐like Kinase1/2
mTOR: mammalian target of rapamycin
PCNA: proliferating cell nuclear antigen
PFK: phosphofructokinase
PK: pyruvate kinase
YAP: yes‐associate protein

## 1 BACKGROUND

The primordial follicle pool does not proliferate after birth and is the sole gametes source in female reproductive life.^1^, ^2^ Therefore, primordial follicle activation is a major biological checkpoint that controls female reproductive potential. Studies have shown that energy levels affect the development of ovaries and follicles.^3^, ^4^, ^5^ Maternal high fat dietary intake during gestation reduced the number of large follicles on day 160 (160 d) and small follicle upon puberty of the offspring in our previous study.^6^ Dietary energy restriction can regulate female reproductive performance and prolong the reproductive lifespan by maintaining the ovarian follicular reserve.^7^, ^8^ However, the specific mechanism by which energy levels affect primordial follicle activation remains unclear.

The AMP‐activated protein kinase (AMPK) found as a highly conserved heterotrimeric kinasein in mammals^9^ is activated by a falling energy status, which promotes adenosine triphosphate (ATP) production by increasing the activity or expression of proteins involved in catabolism and saves ATP by switching off biosynthetic pathways.^10^ Some studies have shown that AMPK activation promotes meiosis and the recovery and maturation of oocytes,^11^, ^12^ but inhibits the development of follicle and granulosa cells.^13^, ^14^ Currently, there are few reports about the effect of AMPK on the activation of primordial follicles.

The Hippo pathway has been extensively studied in mammals in the last several years^15^, ^16^ and its main functions are to regulate cell proliferation, limit tissue growth, and maintain organism homeostasis.^17^, ^18^ The three main components of the Hippo pathway in mammals is a kinase cascade, including the mammalian Sterile20‐like Kinase1/2 (MST1/2), large tumor suppressor1/2 (LATS1/2) and YAP (yes‐associated protein).^19^ It appears the Hippo signaling pathway is not merely related to oocyte polarization,^20^ follicle stem cell maintenance,^21^ but also involved in follicle activation and development.^22^ The Hippo signaling pathway is involved in the serine/threonine protein kinase regulation of primordial follicle activation that is accompanied by a decrease in p‐YAP/YAP protein level.^23^ AMPK activated by energy stress directly phosphorylates YAP at its sites including Ser127 or Ser94, and this phosphorylation induces the combination between YAP and 14‐3‐3 protein or interferes with the interaction between YAP and transcriptional enhanced associate domain protein, thus leading to inhibition of cell growth by repressing YAP target genes.^24^, ^25^, ^26^

The tuberin/tuberous sclerosis complex (TSC)/mammalian target of rapamycin (mTOR) signaling pathway is important to balance the quiescence and activation of primordial follicles,^27^ in which the TSC1/TSC2 heterodimeric complex suppresses the activation of mTOR complex1 (mTORC1) through a GTPase activating protein domain located in TSC2. This leads to suppressed phosphorylation of p70 ribosomal protein S6 kinase 1 (S6K1)‐S6K1‐ribosomal protein S6 (rpS6), eukaryotic translation initiation factor 4B (eIF4B), and eukaryotic initiation factor 4E (eIF4E)‐binding protein 1 (4E‐BP1), thus negatively regulating protein synthesis and primordial follicle activation.^28^, ^29^ AMPK phosphorylates TSC2 and Raptor (a component of mTORC1 that functions as a scaffolding protein) inhibiting the mTORC1 pathway and maintaining ATP levels in cells under energy stress.^30^ Negative regulation of mTORC1 by AMPK is critical to save energy for regulating cell survival and growth under energetic stress.^9^ These studies have shown that energy stress induced by glucose starvation activates AMPK and participates in a variety of cellular processes by regulating Hippo and mTOR signaling pathways. However, whether glucose can affect primordial follicle activation through the above pathways has not been reported.

Therefore, the mouse ovary was used as the model in this study. First, a culture system of mouse ovary in vitro was established to investigate the impact of glucose levels on the activation of the primordial follicle in vitro. On this basis, the signal pathway of glucose affecting the activation of the primordial follicle in vitro and in vivo was further explored.

## 2 MATERIALS AND METHODS

### 2.1 Animals

SPF‐KM (Specific Pathogen Free, Kun Ming) mice were purchased from the Dossy Experimental Animals Co. LTD (Chengdu, China) and housed in the animal facility of Sichuan Agricultural University under a 12 h light/dark cycle with free access to water and food. All procedures were performed in conformity to the Institutional Animal Care and Research Committee of Sichuan Agricultural University (SICAU‐2015‐034).

### 2.2 Effects of glucose levels on the primordial follicle activation in the cultured ovary in vitro

Mouse ovary culture was performed according to the previous description.^31^ Briefly, ovary from 3‐day‐old newborn female mice was collected and isolated in Dulbecco's modification of Eagle's medium (DMEM, Gibco) without glucose and sodium pyruvate supplemented with 3 mg/mL bovine serum albumin (BSA, Sigma) and 1% penicillin‐streptomycin (Sigma). The whole ovary was cultured in DMEM without glucose and sodium pyruvate containing 3 mg/mL BSA (Sigma), 1% penicillin‐streptomycin (Sigma), and 1% Insulin‐Transferrin‐Selenium (Gibco) on hydrophilic polycarbonate membranes (Millipore) floating in 500 μL of culture medium, 12‐well plates (Nunc). To determine the follicles developing ability while cultured in vitro, ovaries from 3‐day‐old newborn mice were cultured for 2 days in vitro and used 3 or 5 days uncultured mouse ovaries as the negative control or the positive control. After the in vitro culture system was successfully established, the ovaries were randomly divided into five treatments according to different glucose concentrations [control (0), 5.6, 25, 50, or 75 mM], and were cultured for 24 or 48 h at 37°C in a humidified atmosphere of 5% CO_2_ followed by histological analysis or protein expression. There were three replicates of per treatment (each replicate in one well of 12‐well plates), and each replicate had four ovaries. Each experiment was performed in triplicate.

### 2.3 Mechanistic effects of glucose on the primordial follicle activation in cultured ovary in vitro

The method of ovary culture in vitro was the same as previous section. Ovaries were cultured with 0 (control) or 25 mM d‐(+)‐glucose for 12, 24, or 48 h to detect protein expression of AMPK‐Hippo‐YAP, AMPK‐YAP, and the mTOR signaling pathways. To investigate the effect of activation or inhibition of AMPK on primordial follicle activation, according to previous studies, ovaries were respectively treated with different concentrations (0, 1, 2 mM or 0, 10, 100 μM,) of 5‐aminoimidazole‐4‐carboxamide 1‐β‐d‐ribofuranoside (AICAR, AMPK activator, catalog S1802, Selleck, USA) or Compound C (AMPK inhibitor, catalog S7306, Selleck, USA) in vitro. To determine the protein changes in the AMPK‐Hippo‐YAP and AMPK‐mTOR signaling pathways, ovaries were incubated with 2 mM AICAR for 4 h^32^ or with 20 μM Compound C for 6 h.^33^ The AICAR and Compound C powder were dissolved in dimethyl sulfoxide (DMSO; catalog D2650, Sigma) and ultrapure water to create a 200 and 10 mM stock, respectively, followed by diluting with culture medium before use. The cultured ovaries were collected for histology or protein expression analysis.

**HEADLIGHTS**

- Glucose is an essential nutrient for the activation of primordial follicles
- The glycolysis pathway is involved in primordial follicle activation of ovaries cultured in vitro
- Glucose affects the primordial follicle activation through the AMPK/mTOR signaling pathway

### 2.4 Effects of glucose on the primordial follicle activation in vivo

The male mice were removed from the litters of 30 single caged SPFKM mice following parturition, leaving only the female offspring. Then 11 litters containing postpartum mice and their newborn female mice (n = 11) were randomly selected. Newborn females were then transferred from the remaining litters to the chosen mice to guarantee litters had 10 newborn females. The newborn mice were allowed to suckle for 4 days and then five of the newborn females in each litter were randomly selected for 24 h fasting (n = 55), and the remaining five were permitted to continue suckling their mother for 24 h (n = 55). On the fifth day of birth, the blood glucose of mice was measured. The mice were then sacrificed with carbon dioxide, and ovaries were collected for histological analysis and protein detection.

### 2.5 Histological and morphological analysis

Cultured ovaries in vitro and treated ovaries in vivo were fixed in 4% paraformaldehyde and subsequently embedded in paraffin then serially sectioned to a thickness of 5 μm. The mounted sections were then stained with hematoxylin and eosin for morphological observation and evaluation of follicle dynamics. The number of follicles at each developmental stage was counted in two consecutive sections that were the largest cross‐section through the center of the ovary, and these counts were subsequently averaged.^34^ Follicles with clearly stained oocyte nucleus were counted to avoid recounting the same follicle. Follicle categories included primordial (oocyte is surrounded by one layer of flattened five to eight somatic cells), primary (oocyte is surrounded with one layer of cuboidal pre‐granulosa cells), and secondary follicle (there are more than one layer of granulosa cells surrounding the oocyte but without an antrum).^34^

### 2.6 Metabolic parameters determination of glycolysis pathway

For the metabolic parameters determination of glycolysis pathway at different glucose levels in cultured ovaries in vitro, commercial assay kits (colorimetry) were used to quantify pyruvate (catalog A081‐1‐1, Nanjingjiancheng, China) and lactic acid (catalog A019‐2‐1, Nanjingjiancheng, China) in the culture medium, and ATP (catalog A095‐1‐1, Nanjingjiancheng, China), hexokinase (HK, catalog A077‐1‐1, Nanjingjiancheng, China), phosphofructokinase (PFK, catalog A129‐1‐1, Nanjingjiancheng, China), and pyruvate kinase (PK, catalog A076‐1‐1, Nanjingjiancheng, China) activity in the ovaries. All of the metabolic parameters analysis was done referring to the manufacturer's instructions, and analyzed in triplicate per sample.

### 2.7 Western blotting

Total protein was extracted by homogenizing the ovaries in WIP (Cell lysis buffer for Western and immunol precipitation, catalog P0013, Beyotime Biotechnology, Shanghai, China) supplemented with a protease inhibitor cocktail (catalog 04693132001, Roche, USA), 1% phenylmethanesulphonyl fluoride (PMSF, catalog ST506, Beyotime Biotechnology, Shanghai, China), and 2% phosphatase inhibitor cocktail A (50×, catalog P1081, Beyotime Biotechnology, Shanghai, China). The supernatant after centrifuging at 12 000 × *g* for 30 min at 4°C, was collected to measure the protein content using a bicinchoninic acid (BCA) protein assay kit (catalog 23227, Thermo Scientific, IL, USA) with a microplate reader, on the basis of manufacturer's protocol. Proteins 20 μg of per sample was electrophoresed on a 10% polyacrylamide gel and was subsequently electrophoretically transferred to polyvinylidene fluoride membranes (catalog 1620177, BioRad, CA, USA). The blots were then incubated with the respective primary antibodies: proliferating cell nuclear antigen (PCNA, catalog 2586T, Cell Signaling Technologies, USA), β‐actin (catalog 4970S, CST), p‐AMPKα (Thr172, catalog 2535S, CST), AMPKα (catalog 2793S, CST), p‐MST1/2 (Thr183/Thr180, catalog GTX54995, Gene Tex, USA), MST1 (catalog 3682S, CST), p‐LATS1 (Thr1079, catalog 8654S, CST), LATS1 (catalog 3477S, CST), p‐YAP (Ser127, catalog 13008S, CST), p‐YAP (Ser94, catalog 254542, ABBIOTEC, USA), YAP (catalog 4912S, CST), p‐mTOR (Ser2448, catalog 2971S, CST), mTOR (catalog 2972S, CST), p‐S6 (Ser235/236, catalog 4858S, CST), S6 (catalog 2217S, CST), and Caspase‐3 (catalog 9665T, CST). Protein signals were detected using ECL western blotting detection reagent (catalog 1705060, BioRad) on a Molecular Imager ChemiDoc XRS+ System (BioRad). Blots were quantified using Image J software (National Institutes of Health, Bethesda, MD, USA) and the levels of β‐actin expression were used as an internal control.

### 2.8 Statistical analysis

All ovary culture in vitro experiments were repeated at least three times, and data are presented as mean ± SEM. One‐way analysis of variance (ANOVA) was applied to analyze the difference among treatment groups using SAS 9.4 (SAS Institute, Inc., Cary, North Carolina) and multiple comparisons between two groups were made according to the Duncan method. T‐test was performed to determine significant difference between the treatment and control groups using Graphpad Prism 5 software (GraphPad Software Incorporated, La Jolla, CA, USA). A value of *P* < .05 was considered statistically significant.

## 3 RESULTS

### 3.1 Effects of different glucose level on the primordial follicle activation

**FIGURE 1:**
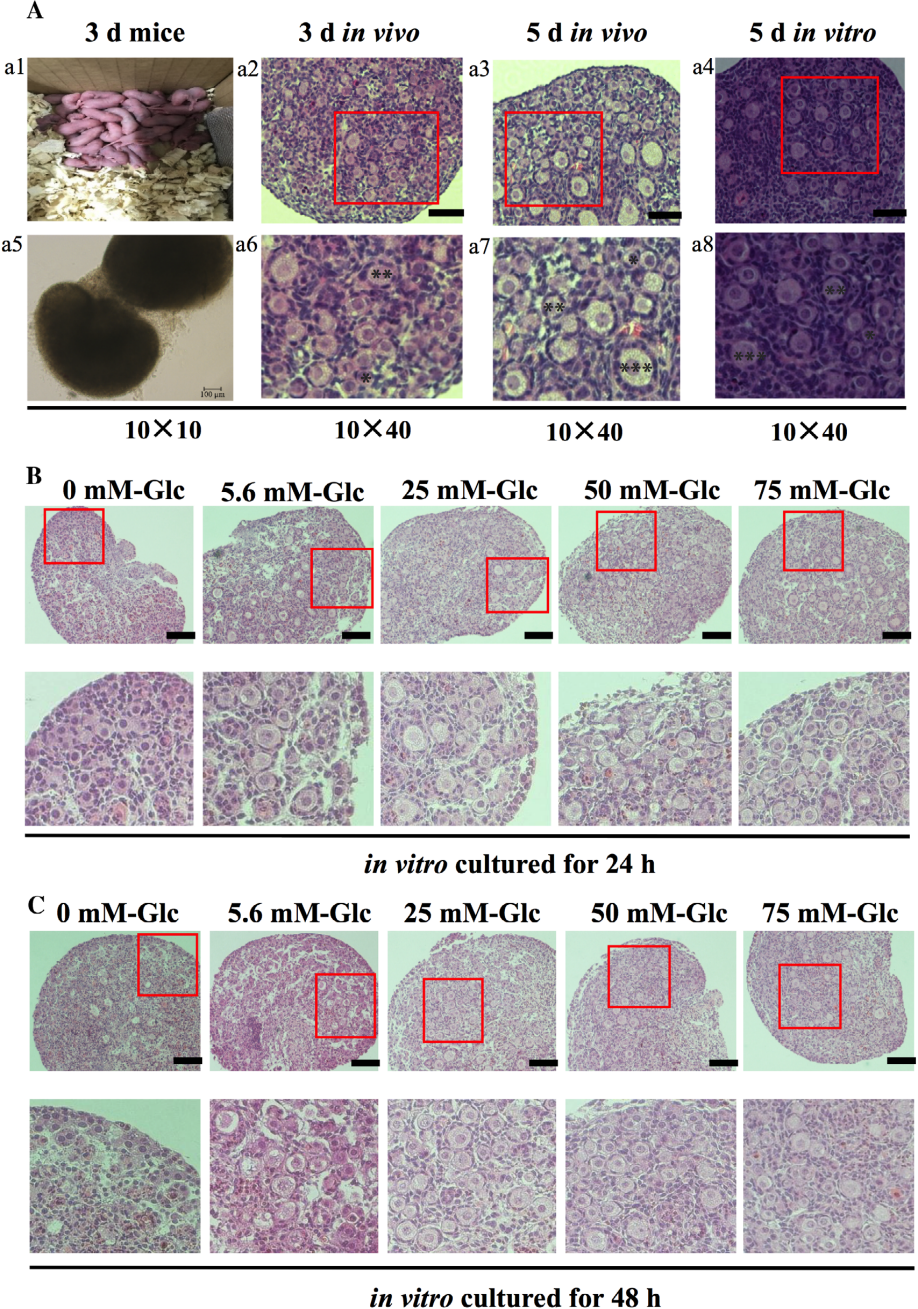
Effects of different glucose concentrations on the activation of primordial follicles in ovaries cultured in vitro. A, Representative images depicting the typical morphology in cultured ovaries. Panel A1 showed the 3 day mice pups and Panel A5 showed the whole ovarian under the microscope, Panel A2 showed the uncultured 3 day mouse ovaries, Panel A3 showed the uncultured 5 day mouse ovaries, Panel A4 showed the 5 days in vitro (3 days old ovaries cultured in vitro 2 days). Scale bars 100 μm, original magnification ×100 (A2‐4) or ×400 (A6‐8). B, Effects of different glucose level (from left to the right: 0, 5.6, 25, 50, and 75 mM) on the proportion of follicles in ovary cultured for 24 h in vitro: scale bars 100 μm, original magnification ×200. C, Effects of different glucose level (from left to the right: 0, 5.6, 25, 50 and 75 mM) on the activation of primordial follicles in ovary cultured for 48 h in vitro: scale bars 100 μm, original magnification ×200. Glc, glucose. ^*^Denotes primordial follicle, ^**^primary follicle, and ^***^denotes secondary follicles

For determining the ability of follicles to continue developing while cultured in vitro, ovaries from 3‐day‐old mice were cultured for 2 days in vitro. We observed similar morphology among the uncultured 3‐day mouse ovaries (Figure 1A(2), negative control), uncultured 5‐day mouse ovaries (Figure 1A(3), positive control) and the 5 day in vitro cultured ovaries (3‐day‐old ovaries cultured in vitro for 2 days, Figure 1A(4)). Furthermore, the ovarian tissue structure remained intact, and follicles had begun activating, suggesting the ovary culture system was successful (Figure 1A). Exposure of the in vitro cultured ovaries to different levels of glucose (0, 5.6, 25, 50, or 75 mM glucose), for either 24 or 48 h, significantly affected the activation of ovarian primordial follicles (Figure 1B,C). The primordial follicles proportion decreased with exposure to glucose, for either 24 or 48 h (*P* < .05, Tables 1 and 2), and the primary follicle proportion increased (*P* < .05) compared with control treatment. No significant difference was observed in the primordial, primary, or secondary follicle proportions among the 25, 50, and 75 mM glucose treated groups, with either 24 or 48 h exposure (*P* > .05). However, when treated with glucose in vitro for 24 h, the proportion of secondary follicles in the 25 mM treatment was higher than that in the 5.6 mM treatment (20.18 ± 0.76% vs 6.99 ± 3.55%, *P* < .05). And in vitro culture in the 25 mM treatment for 48 h resulted in the primary/primordial follicles’ ratio was higher than that in the 5.6 mM treatment (2.29 ± 0.34 vs 1.06 ± 0.28, *P* < .05). The above results showed that glucose was an essential nutrient for the primordial follicle activation and 25 mM glucose concentration was the optimal level for the primordial follicle activation identified in this study.

**TABLE 1:** Effects of different glucose level on the proportion of follicles in ovary cultured for 24 h in vitro

	0 mM	5.6 mM	25 mM	50 mM	75 mM	*P‐*value
Primordial follicle (%)	76.36 ± 4.52^a^	54.51 ± 4.13^b^	39.30 ± 10.16^b^	38.82 ± 3.47^b^	36.59 ± 2.99^b^	.008
Primary follicle (%)	17.49 ± 3.04^b^	38.49 ± 4.05^a^	40.52 ± 9.86^a^	43.40 ± 1.38^a^	48.05 ± 2.44^a^	.049
Secondary follicles (%)	6.15 ± 4.24^c^	6.99 ± 3.55^bc^	20.18 ± 0.76^a^	17.78 ± 4.84^ab^	15.36 ± 3.82^abc^	.045
Primary/primordial	0.28 ± 0.05	0.79 ± 0.14	1.92 ± 1.21	1.35 ± 0.19	1.34 ± 0.12	.282

Different lowercase letters in the same row indicate significant differences (*P* < 0.05), n = 6.

**TABLE 2:** Effects of different glucose level on the proportion of follicles in ovary cultured for 48 h in vitro

	0 mM	5.6 mM	25 mM	50 mM	75 mM	*P‐*value
Primordial follicle (%)	98.02 ± 1.98^a^	43.58 ± 4.11^b^	26.96 ± 3.32^c^	29.36 ± 3.24^c^	27.57 ± 2.08^c^	<.01
Primary follicle (%)	1.98 ± 1.98^c^	43.97 ± 7.99^b^	57.80 ± 2.79^ab^	52.31 ± 4.18^ab^	62.46 ± 0.07^a^	.031
Secondary follicles (%)	0.00 ± 0.00^b^	12.46 ± 5.38^a^	15.25 ± 3.97^a^	18.33 ± 3.13^a^	9.97 ± 2.10^ab^	.031
Primary/primordial	0.02 ± 0.02^c^	1.06 ± 0.28^b^	2.29 ± 0.34^a^	1.99 ± 0.31^a^	2.35 ± 0.24^a^	<.01

Different lowercase letters in the same row indicate significant differences (*P* < .05), n = 6.

### 3.2 Effects of different glucose concentrations on total protein content and PCNA expression of ovaries cultured in vitro

**FIGURE 2:**
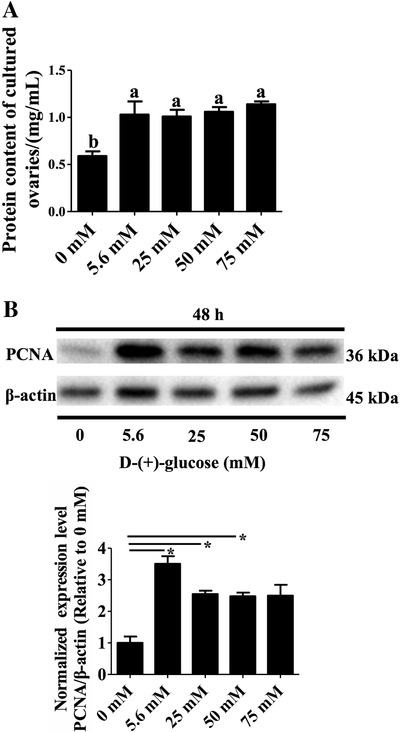
Effects of different glucose concentrations on total protein content and PCNA expression of ovary cultured in vitro. A, Effects of different glucose concentrations on total protein content of ovary cultured in vitro: the number of ovaries in the control and glucose treatment groups was the same (n = 12). B, Effects of different glucose concentrations on PCNA expression of ovary cultured in vitro

Compared with the control (0 mM) the total protein content of ovaries in each glucose treatment was significantly higher (1.01 ± 0.07 mg/mL to 1.14 ± 0.03 mg/mL vs 0.59 ± 0.05 mg/mL, *P* < .05, Figure 2A), but no significant difference was observed among different glucose levels (*P* > .05). Similarly, the exposure to glucose for 48 h at 5.6, 25, or 50 mM concentrations resulted in increased PCNA levels (*P* < .05, Figure 2B). The above results indicate that the addition of the energy substrate glucose, in vitro, led to an increased overall growth of the cultured ovaries.

### 3.3 Effects of different glucose concentrations on the metabolite content of the glycolysis pathway

**FIGURE 3:**
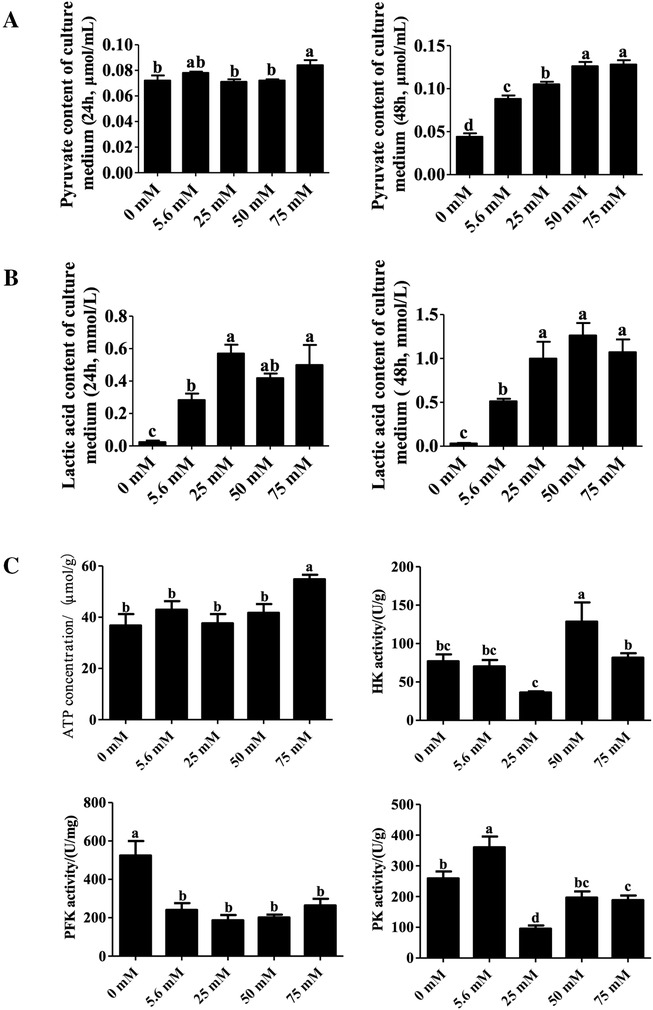
Effects of glucose on metabolite content of the glycolysis pathway. A, Effects of different glucose level on pyruvate content in culture medium of ovary cultured in vitro (left, cultured for 24 h; right, cultured for 48 h): the number of ovaries in the control and glucose treatment groups was the same. B, Effects of different glucose level on lactic acid content in culture medium of ovary cultured in vitro: (left, cultured for 24 h; right, cultured for 48 h): the number of ovaries in the control and glucose treatment groups was the same. C, Effects of different glucose level on ATP concentration, HK, PFK, and PK activity of ovary cultured in vitro for 48 h: the number of ovaries in the control and glucose treatment groups was the same. n = 12. Abbreviation: HK, hexokinase; PFK, phosphofructokinase; PK, pyruvate kinase

Following in vitro culture for 24 h, the pyruvate content in the cell culture medium of the 75 mM glucose treatment was higher than that of the control, as well as the 25 and 50 mM glucose treatment groups (*P* < .05, Figure 3A). The lactic acid content of the culture medium in each glucose treatment was higher than that in the control (*P* < .05, Figure 3B), and lactic acid content of the culture medium in the 25 and 75 mM glucose treatment was higher than that in the 5.6 mM glucose treatment (*P* < .05). Following in vitro culture for 48 h, the pyruvate content of the cell culture medium in each glucose treatment was higher than that in the control (*P* < .05, Figure 3A). The pyruvate content significantly increased in a dose‐dependent manner between the 0, 5.6, 25, and 50 mM groups (*P* < .05, Figure 3A). The lactic acid content of the cell culture medium in each glucose treatment was higher than that in the control (*P* < .05, Figure 3B).

Following in vitro culture for 48 h, the quantity of ovarian ATP in the 75 mM glucose treatment was higher than that in the untreated control group and in the other glucose treatments (*P* < .05, Figure 3C). The ovarian HK activity of the 50 mM glucose treatment was higher than that of the control and other glucose treatments (*P* < .05, Figure 3C). The ovarian PFK activity was higher in the control when compared to the glucose treatments (*P* < .05, Figure 3C). Conversely, ovarian PK activity was significantly higher in the 5.6 mM glucose treatment than in the control and the other glucose treatments (*P* < .05, Figure 3C).

The above results provide evidence that the glycolysis pathway in the ovarian cells is altered by glucose treatment and may play a role in the primordial follicle activation in vitro.

### 3.4 Effects of glucose on AMPK‐Hippo‐YAP, AMPK‐YAP, and mTOR signaling pathway protein expression in ovaries cultured in vitro

**FIGURE 4:**
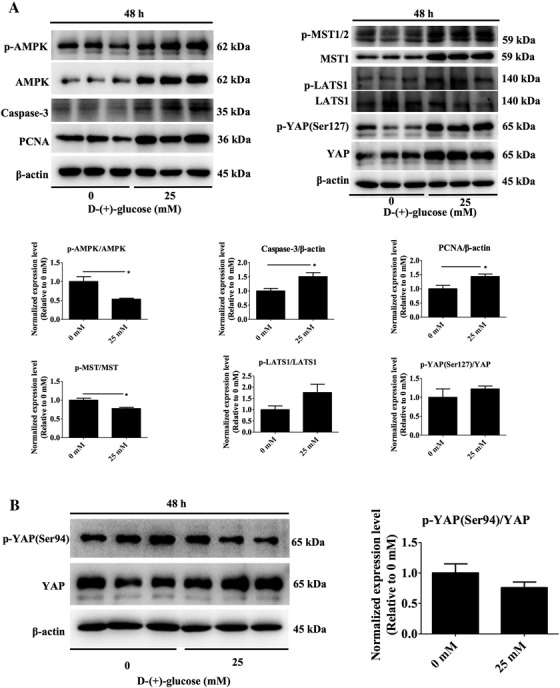
Effects of glucose on AMPK‐Hippo‐YAP and AMPK‐YAP signaling pathway protein expression in ovaries cultured for 48 h in vitro

There were no significant differences in AMPK‐Hippo‐YAP signaling pathway protein expression in ovaries cultured in vitro for 12 and 24 h. (Data not shown, *P* > .05). However, in ovaries cultured without glucose for 48 h ovarian p‐AMPK/AMPK and p‐MST/MST were higher (*P* = .024, *P* = .023; Figure 4A), and Caspase‐3 and PCNA were lower (*P* = .039, *P* = .043), while p‐LATS1/LATS1 and p‐YAP(Ser127)/YAP showed no significant differences when compared with the 25 mM glucose treated ovaries (*P* = .136, *P* = .401).

The exposure of ovaries to 25 mM glucose for 48 h in vitro resulted in a significant decrease in p‐AMPK/AMPK protein levels (*P* = .024, Figure 4A). However, no difference was observed in the protein levels of p‐YAP(Ser94)/YAP between the two treatments (*P* = .243, Figure 4B).

**FIGURE 5:**
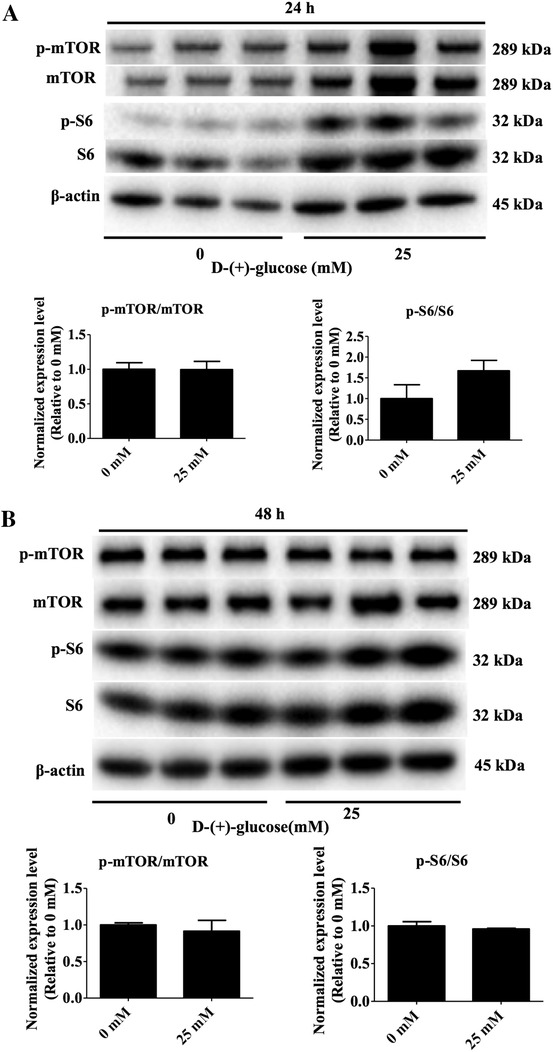
Effects of glucose on mTOR signaling pathway protein levels in ovaries cultured for 24 h (A) and 48 h (B) in vitro

There was no difference in p‐mTOR/mTOR and p‐S6/S6 protein levels in ovaries treated with 0 mM glucose when compared to ovaries exposed to 25 mM glucose, for either 24 or 48 h (*P* > .05, Figure 5A,B).

### 3.5 Effects of activation of AMPK on the primordial follicle activation and protein expression of ovaries cultured in vitro

**FIGURE 6:**
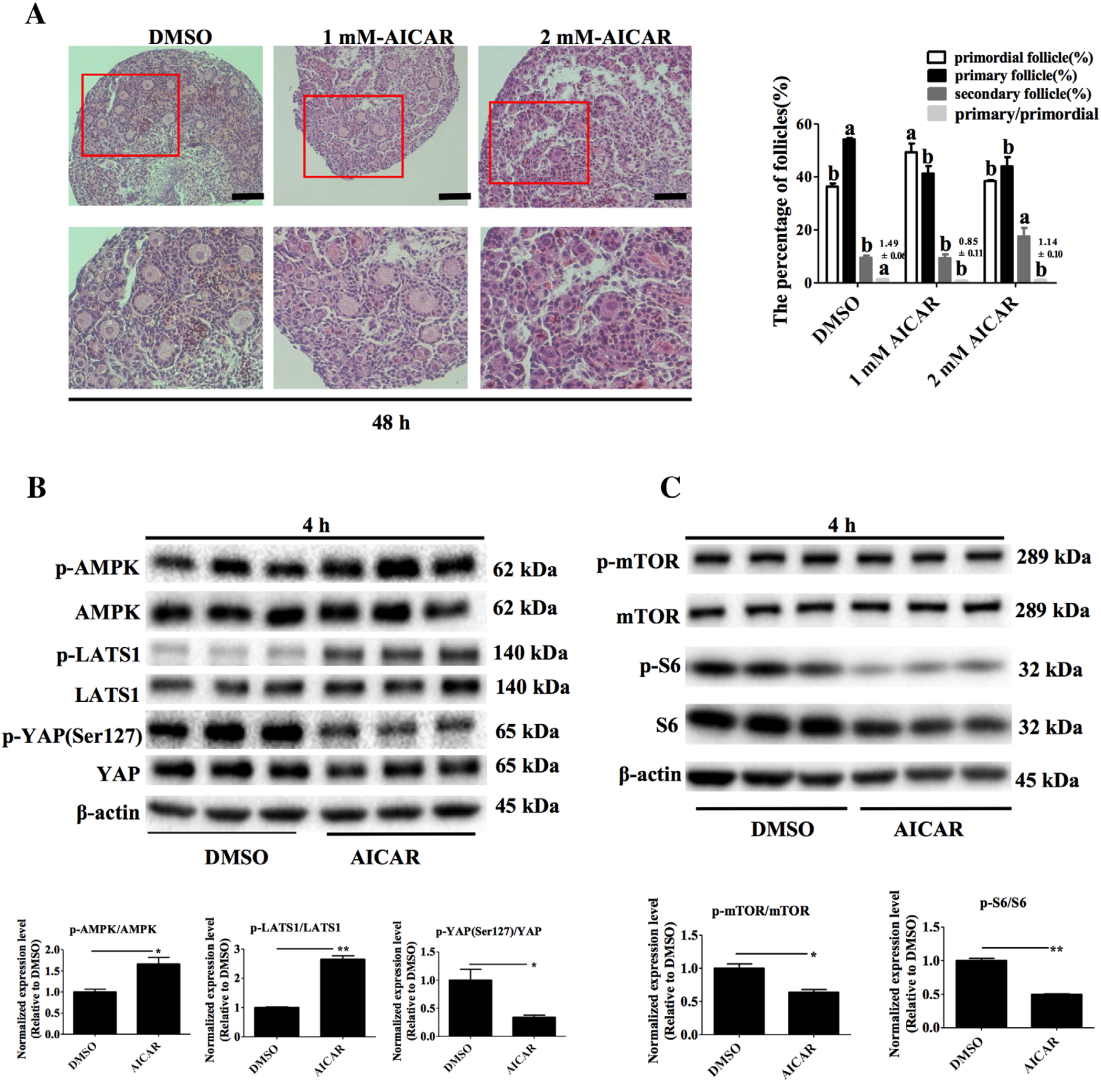
Mediatory role of activation of AMPK in the activation of primordial follicle in vitro. A, Mouse ovaries were cultured with AICAR for 48 h and were stained with H&E (left panel) to detect the proportion of follicle types in ovaries (right panel). Scale bars 100 μm, original magnification ×200, bars with different letters are significantly different (*P* < .05). B, Effects of activation of AMPK on AMPK‐Hippo‐YAP signaling pathway protein levels in ovaries cultured in vitro. C, Effects of the activation of AMPK on AMPK‐mTOR signaling pathway protein levels in ovaries cultured in vitro

The differences of primordial follicle activation were significant when ovaries were treated with different concentrations of the AMPK activator AICAR for 48 h (Figure 6A, left panel). The proportion of primordial follicles in the 1 mM AICAR treatment was higher than that in the control (untreated group) and in the 2 mM AICAR treatment (*P* < .05, Figure 6A right panel). The primary follicle proportion and primary/primordial follicles ratio in the untreated control group were higher than that in the 1 mM AICAR and 2 mM AICAR treatment groups (*P* < .05). The secondary follicle proportion in the 2 mM AICAR treatment was higher than that in the control (untreated group) and the 1 mM AICAR treatment (*P* < .05).

Ovaries incubated with 2 mM AICAR for 4 h caused a significant increase in p‐AMPK/AMPK and p‐LATS1/LATS1 protein levels (*P* < .05, *P* < .01, Figure 6B) and a significant decrease in p‐YAP (Ser127)/YAP (*P* = .029, Figure 6B). We also found a decrease in p‐mTOR/mTOR (*P* = .010, Figure 6C) and p‐S6/S6 protein levels (*P* < .01, Figure 6C).

### 3.6 Effects of the inhibition of AMPK on the primordial follicle activation and protein levels of ovaries cultured in vitro

**FIGURE 7:**
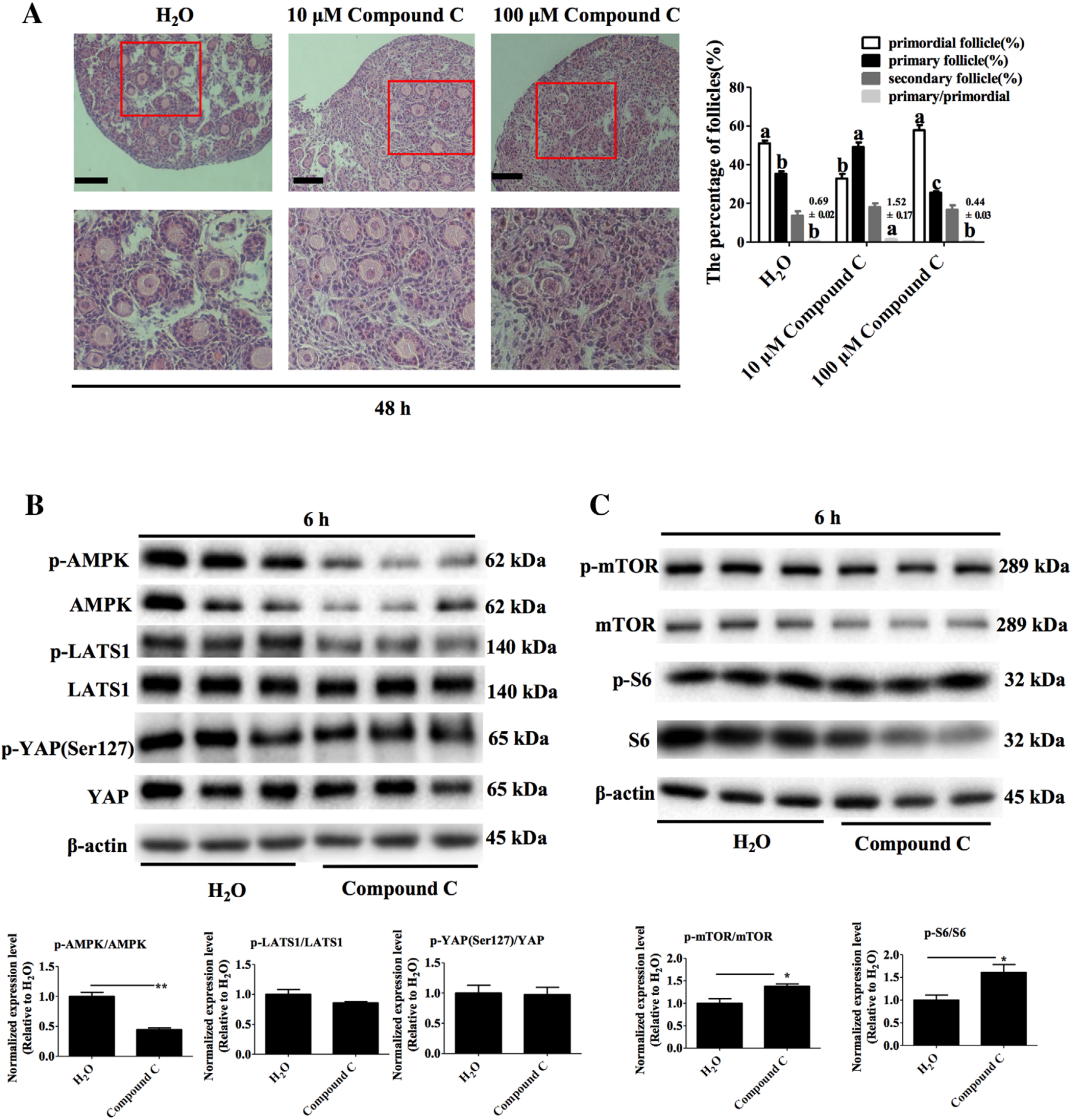
Mediatory role of inhibition of AMPK in the activation of primordial follicle in vitro. A, Mouse ovaries were cultured with Compound C for 48 h and were stained with H&E (left panel) to detect the proportion of follicle types in ovaries (right panel). Scale bars 100 μm, original magnification ×200, bars with different letters are significantly different (*P* < .05). B, Effects of the inhibition of AMPK on AMPK‐Hippo‐YAP signaling pathway protein levels in ovaries cultured in vitro. C, Effects of the inhibition of AMPK on AMPK‐mTOR signaling pathway protein levels in ovaries cultured in vitro

Histology results showed significant differences in primordial follicle activation amongst ovaries that were treated with different concentrations of the AMPK inhibitor, Compound C for 48 h (Figure 7A left panel). The 10 μM Compound C treatment had a lower primordial follicle percentage than the untreated control group or the 100 μM Compound C treatment (*P* < .05, Figure 7A, right panel). The primary follicle percentage and the ratio of primary/primordial follicles in 10 μM Compound C treatment were also higher than those in the control or the 100 μM Compound C treatment (*P* < .05).

Ovaries treated with 20 μM Compound C for 6 h showed an extremely significant reduction of p‐AMPK/AMPK protein levels (*P* = .002, Figure 7B) but no insignificant differences in p‐LATS1/LATS1 and p‐YAP(Ser127)/YAP when compared to the control (*P* = .173, *P* = .892). On the other hand, compared with the control group p‐mTOR/mTOR and p‐S6/S6 protein levels increased (*P* = .029, *P* = .043, Figure 7C).

The above results indicated that glucose affects the primordial follicle activation in vitro through AMPK/mTOR, rather than AMPK‐Hippo‐YAP, signaling pathway.

### 3.7 Effects of glucose on the ovarian primordial follicle activation in vivo

**FIGURE 8:**
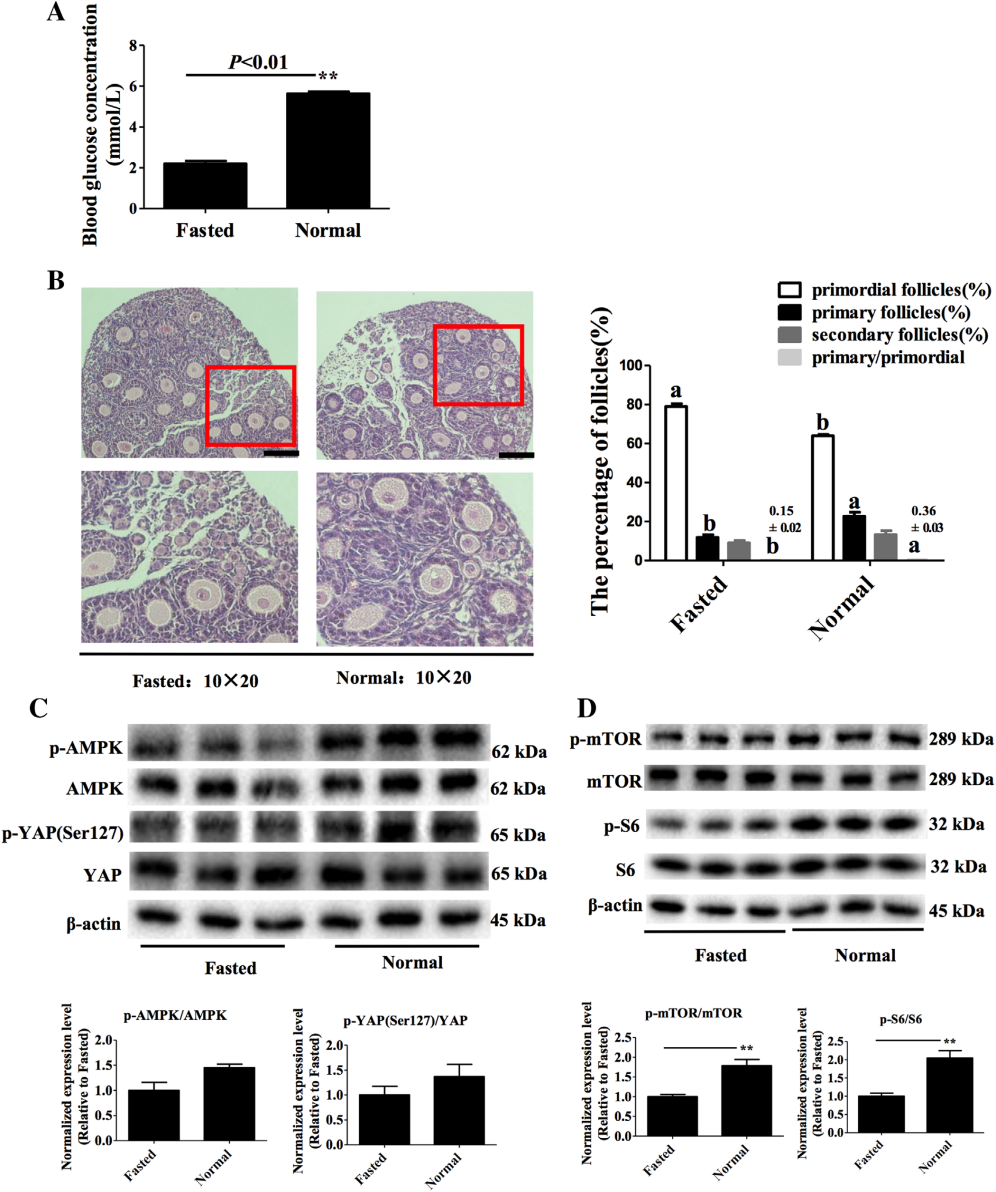
Effects of glucose on the activation of ovarian primordial follicles in vivo. A, Effects of fasting on blood glucose concentrations: control (normal breastfed for 24 h), 5.64 ± 0.10 mM, n = 55; treatment (starvation for 24 h), 2.20 ± 0.13 mM, n = 58; mean ± standard error. B, Effects of fasting on the activation of primordial follicles. The ovaries were stained with H&E (left panel) to detect the proportion of follicle types in ovaries (right panel). Scale bars 100 μm, original magnification ×200, bars with different letters are significantly different (*P* < .01, n = 8). C, Effects of fasting on AMPK‐Hippo‐YAP signaling pathway protein expression in ovaries. D, Effects of fasting on AMPK‐mTOR signaling pathway protein expression in ovaries

After fasting for 24 h, the blood glucose levels of newborn 4‐day female mice was lower than that of normal breastfed newborn 4‐day female mice (5.64 ± 0.10 mM, n = 55 vs 2.20 ± 0.13 mM, n = 55; *P* < .01, Figure 8A).

Reduced blood glucose caused by fasting for 24 h inhibited primordial follicle activation in the 4‐day mice (Figure 8B, left panel). The primordial follicle proportion of fasted newborn 4‐day mice was significantly higher than that of normal breastfed newborn 4‐day mice (*P* < .01, Figure 8B, right panel). However, the proportion of primary follicles and the ratio of primary/primordial follicles were significantly lower than that of normal breastfed newborn 4‐day mice (*P* < .01).

After fasting for 24 h, ovarian p‐AMPK/AMPK and p‐YAP(Ser127)/YAP protein levels had no significant difference from that of the normal breastfed group (*P* = .061, *P* = .293, Figure 8C), but p‐mTOR/mTOR and p‐S6/S6 protein levels were significantly reduced (*P* = .009, *P* = .009, Figure 8D).

The above results showed that nutritional levels affect the activation of primordial follicles via the mTOR signaling pathway, in vivo.

## 4 DISCUSSION

Primordial follicle activation and development involves a complex network of factors regulated by endocrine signaling and intracellular signaling pathways.^35^ Ewes infused with glucose by jugular cannulae had a significantly increased number of follicles with a diameter >1 mm.^13^ It also found that improving the blood glucose of ewes to appropriate levels through diet could increase the number of follicles and the developmental competence of the oocytes.^36^ In our study, we found that the primordial follicle proportion decreased with different levels of glucose and the primary follicles’ proportion increased. Furthermore, the total protein content and PCNA expression of ovaries exposed to glucose were higher than in ovaries without access to glucose. PCNA expression is considered as a reliable marker for primordial follicle activation.^1^, ^37^ Consistent with previous studies, our results suggest that the addition of the energy substance glucose increases ovarian follicle growth and development in vitro. Glucose is a major energy source for whole cultured ovaries and for isolated follicles at the pre‐ovulatory stage of development.^38^ Oocyte can directly use pyruvate but not lactic acid or glucose during in vitro maturation, however the cumulus cells are able to metabolize lactic acid or glucose and supply the oocyte with products that permit maturation.^39^ Pre‐antral ovarian follicles of pre‐pubertal female mice utilize a predominantly and steadily glycolytic method of ATP production, which 24‐60% of total glucose is consumed by glycolysis and glucose consumption along with lactic acid production increase with in vitro development.^40^ This study was consistent with previous reports in that the metabolic indicators of glycolysis were significantly different at the five glucose levels tested. We initially came to the conclusion that glucose was an essential nutrient for the primordial follicle activation and that 25 mM glucose was the optimal level for primordial follicle activation. Moreover, we concluded that the glycolysis pathway was involved in the primordial follicle activation in vitro.

There are two parallel signal pathways in which energy stress activates AMPK to regulate YAP activity in cells. One of them is the AMPK‐AMOTL1‐Lats1‐YAP(Ser127) pathway and the other is the AMPK‐YAP(Ser94) pathway.^41^ It has been shown that the phosphorylation of YAP at Ser127 is the major phosphorylation site regulated by the Hippo pathway and increased YAP localized in the cytoplasm in glucose‐starved HEK293A cells, and the phosphorylation of YAP at Ser127 markedly decreased when glucose was added back to these glucose‐deprived cells.^26^ The AMPK activators AICAR, metformin, and phenformin inhibited YAP nuclear localization and increased p‐YAP(Ser127)/YAP protein expression level of HEK293A, HaCaT, and MEFs cells.^24^ YAP phosphorylation induced by energy stress was also found in myoblast C2C12, mammary epithelial MCF10A, and the cervical cancer HeLa cell lines.^25^ We found that although p‐AMPK/AMPK and Hippo upstream p‐MST/MST increased in glucose‐deprived ovaries, p‐LATS1/LATS1, and downstream p‐YAP(Ser127)/YAP, p‐YAP(Ser94)/YAP showed no difference compared with the 25 mM glucose treatment. The effector YAP of the Hippo pathway was not significantly affected even though AMPK was activated in glucose‐deprived ovaries, which was inconsistent with previous studies on cells. We speculate that the reasons for the inconsistent results may include: (a) There should be some differences in the growth mode, energy metabolism, and energy stress sensitivity *in vitro* of the different types of cells used by predecessors and the ovarian tissue in this study; (b) The exact function and regulatory mechanism of the Hippo signaling pathway in different tissues, organs, and cells show the same or different characteristics.^42^, ^43^, ^44^, ^45^

We subsequently found that 1 mM AICAR treatment significantly increased the primordial follicle proportion and decreased the primary follicle proportion and the primary/primordial follicle ratio. Interestingly, the opposite was true in ovary treated with 10 μM Compound C that was consistent with a previous report regarding the positive effect of Compound C on follicle activation.^46^ Previously, we have shown that the phosphorylation level of AMPK significantly increased in ovaries treated with adiponectin, but the primary follicle number and the primary/primordial follicle ratio significantly decreased.^33^ It is worth noting that p‐LATS1/LATS1 and p‐YAP(Ser127)/YAP protein levels were not significantly changed when AMPK was suppressed, however p‐LATS1/LATS1 increased significantly while p‐YAP(Ser127)/YAP decreased significantly when AMPK was activated, and this needs to be further studied.

Reduced levels of both total and phosphorylated AMPK while increased levels in phosphorylation of both mTOR and its downstream target p70S6K were observed in AMPK directed siRNA‐transfected U251 cells.^47^ Glucose withdrawal rapidly activated AMPK, resulting in continuous inhibition of p70S6K activity, thus increased the levels of phosphorylation‐acetyl coenzyme A carboxylase (p‐ACC) in HCE‐T cells. And the same treatment decreased the levels of pS6 in Calu‐3 and H441 cells.^48^ In MEFs cells treated with metformin or phenformin, the p‐AMPK (Thr172) and p‐ACC(Ser79) levels were upregulated, while the level of p‐S6K1(Thr389), downstream of mTOR, was downregulated.^49^ Negative regulation of the mTOR signaling pathway by AMPK has been seen in different types of cells including Calu‐3, H441, HCE‐T, H4IIE, MEFs, hepatocyte, and MIN6 cells treated with AICAR.^48^, ^50^ Our results and those of the above reports consistently indicate that AMPK activation directly inhibits the mTOR signaling pathway, and the effect of AICAR on AMPK/mTOR appears to be stronger than that of glucose starvation. Moreover, p‐TSC2/TSC2 significantly decreased, p‐mTOR/mTOR, p‐S6, and p‐eIF4B significantly increased in 10 days old CD‐1 mouse ovaries incubated with 10 μM Compound C.^46^

**FIGURE 9:**
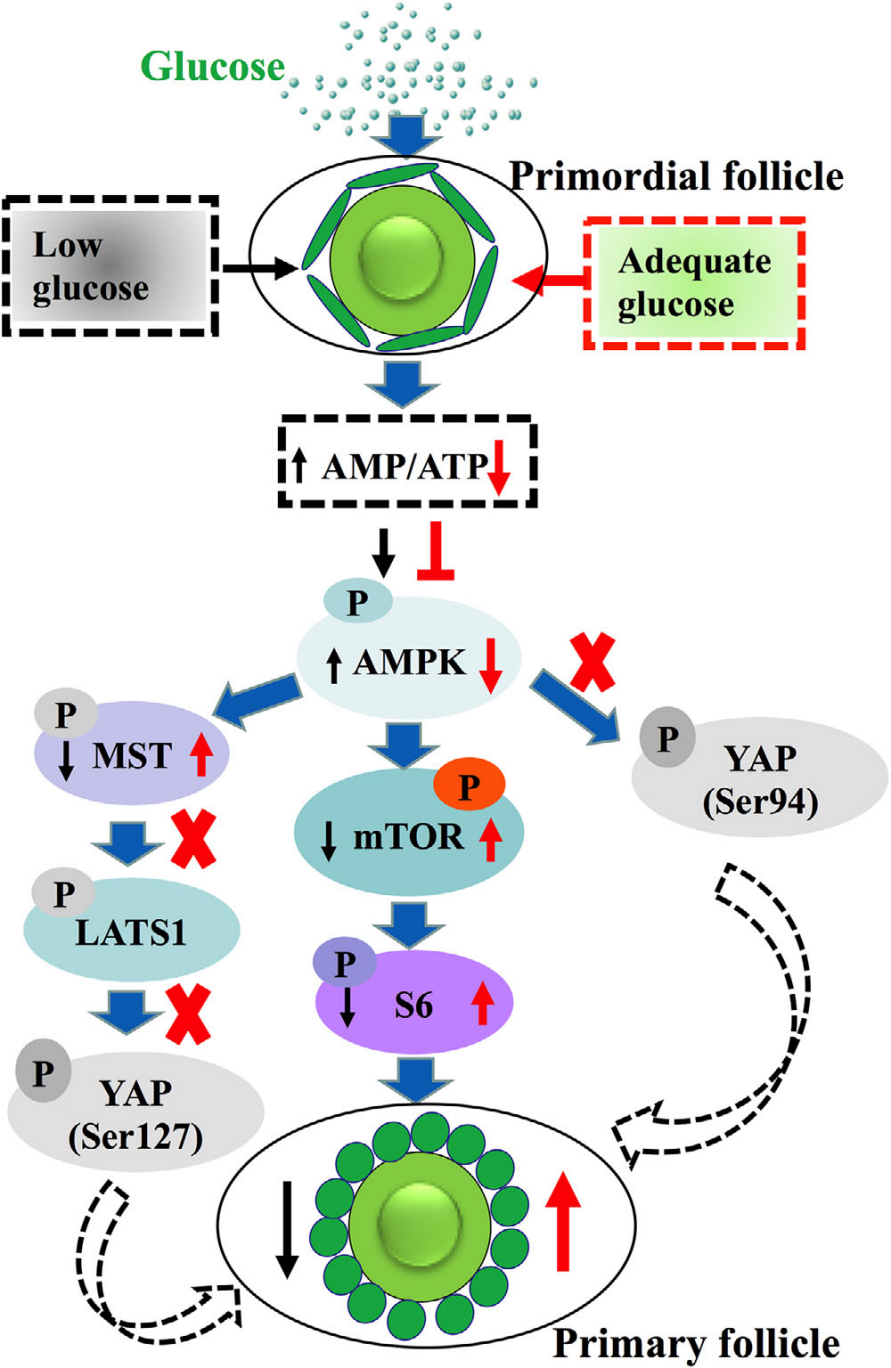
A model for glucose regulation ovarian primordial follicles activation via the AMPK/mTOR signaling pathway. Only core components of the pathway are shown. Adequate glucose (middle right) reduce the ratio of AMP/ATP in primordial follicle, thus inhibit phosphorylation of AMPK. In turn, it promotes the phosphorylation of mTOR and activates the phosphorylation of S6 protein. By activating the expression of p‐S6 downstream genes and proteins, the primordial follicle is activated finally. The AMPK‐Hippo‐YAP (bottom left) and AMPK‐YAP (bottom right) signaling pathway does not work in the ovarian primordial follicles activation. Black arrow shows the effect of low glucose, the red arrow shows the effect of adequate glucose. The red cross symbol means no effect

In order to further clarify the effects of glucose on primordial follicle activation, we examined the ovaries of 4 days old mice that had either been fasted or allowed to breastfeed for a 24 h period. We found that the blood glucose levels of 4 days female mice fasted for 24 h was lower than that of normal breastfed 4 d female mice. This phenomenon was in accordance with previous research showing the effective reduction of blood glucose in rats or mice caused by fasting for 24 h or more than 24 h.^51^, ^52^, ^53^ The follicles grow from the primordial to primary and secondary stages independent of pituitary gonadotropins,^2^, ^34^, ^54^ therefore it is unlikely that hormonal changes caused by fasting influence the primordial follicle activation. It has been previously shown that in female mice fasting for 48 or 64 h that serum glucose concentrations significantly decreased, follicles began to degenerate, oocytes in the follicles became unviable, and the number of cumulus‐oocyte complexes (COCs) harvested significantly decreased.^51^ Our in vivo results showed an inhibition of primordial follicle activation coinciding with a decrease in blood glucose (Figure 9) and were consistent with previous reports that improving blood glucose could promote follicular development.^13^, ^36^ Previously, it has been shown that the levels of p‐AMPK/AMPK protein significantly increased while the expression of p‐mTOR, mTOR protein significantly decreased in rats with a 40% diet restriction or metformin compared with control rats fed ad libitum.^55^ Calorie restriction, glucose deprivation, or 2‐DG trigger metabolic stress that activates AMPK, which activates the TSC by phosphorylating certain sites on tuberin to enhance TSC's activity. TSC inhibits mTOR by facilitating Rheb GTP dephosphorylation, eventually inhibition of mTOR reduces protein synthesis and cell growth/proliferation.^56^ The mTOR signaling pathway was inhibited by energy stress caused by starvation, which would repress the mRNA expression of *Hif1α* and *Vegfa* in its downstream genes,^46^ negatively regulate the protein synthesis, granulosa cell, and oocyte growth,^28^, ^29^ and finally inhibit primordial follicle activation.

To summarize, we have shown evidence that glucose is an essential nutrient for the activation of primordial follicles, and 25 mM glucose is the optimal level for the in vitro activation of the primordial follicle in this study. The glycolysis pathway is involved in primordial follicle activation of ovary cultured in vitro. Glucose affects the primordial follicle activation through the AMPK/mTOR signaling pathway.

## AUTHOR CONTRIBUTIONS

SX, XW, and MX conceived of and designed the study. XW, YD, MX, ZL, YZ, and SC performed the animal experiment and biological experiments. XW, SX, and YD completed statistical analysis. SX and XW wrote the manuscript. All authors critically reviewed the manuscript and gave final approval for the version to be published.

## AVAILABILITY OF DATA AND MATERIALS

The data sets used in the current study are available from the corresponding author on reasonable request.

## CONFLICT OF INTEREST

The authors declare that there is no conflict of interest.
